# Clinical Variability and Genotype Driven Outcomes in an International Cohort of 14 Pediatric *FIG4* Patients

**DOI:** 10.1002/acn3.70561

**Published:** 2026-11-03

**Authors:** Andrea Gangfuß, Vykuntaraju K. Gowda, Daniel Natera‐de Benito, Pinki Munot, Maria Vanegas, Alejandra Darling, Constanze Reutlinger, Laura Carrera‐Garcia, Gilbert Wunderlich, Heike Kölbel, Burkhard Stüve, David Muhmann, Lucía Monfort Belenguer, Diana‐Gabriela Barca, Viveka Santhosh Reddy C, Varunvenkat M. Srinivasan, Cristina Molina Anguita, Heinz Jungbluth, Hormos Salimi Dafsari, Thomas Rossor, Johann Böhm, Ulrike Schara‐Schmidt, Andreas Roos

**Affiliations:** ^1^ Department of Pediatric Neurology University Duisburg‐Essen Essen Germany; ^2^ Department of Pediatric Neurology Indira Gandhi Institute of Child Health Bangalore India; ^3^ Neuromuscular Unit, Neurology Department Hospital Sant Joan de Déu Barcelona Spain; ^4^ Dubowitz Neuromuscular Centre, Great Ormond Street Hospital for Children London UK; ^5^ Children's Neurosciences Evelina London Children's Hospital at Guy's and St Thomas' National Health Service Foundation Trust London UK; ^6^ Metabolic Unit, Movement Disorders Unit, Neurology Department Hospital Sant Joan de Déu Barcelona Spain; ^7^ Department of Pediatric Neurology DRK‐Kinderklinik Siegen gGmbH Siegen Germany; ^8^ Department of Neurology and Center for Rare Diseases, Faculty of Medicine University Hospital Cologne Cologne Germany; ^9^ Pediatric Neurology Clínic Universitary Hospital of Valencia Valencia Spain; ^10^ Pediatric Neurology Department, Center of Expertise for Rare Diseases in Pediatric Neurology “Prof. Dr. Alexandru Obregia” Clinical Psychiatry Hospital Bucharest Romania; ^11^ Carol Davila University of Medicine and Pharmacy Bucharest Romania; ^12^ Pediatric Neurology Consorci Sanitari de Terrassa Barcelona Spain; ^13^ Faculty of Life Sciences and Medicine Randall Centre for Cell and Molecular Biophysics, King's College London London UK; ^14^ Department of Pediatrics and Center for Rare Diseases, Faculty of Medicine and University Hospital Cologne University of Cologne Cologne Germany; ^15^ Max‐Planck‐Institute for Biology of Ageing, Cologne Excellence Cluster on Cellular Stress Responses in Aging Associated Diseases (CECAD) Cologne Germany; ^16^ Department of Translational Medicine and Neurogenetics Institute of Genetics and Molecular and Cellular Biology (IGBMC), INSERM U1258, CNRS UMR7104, University of Strasbourg Illkirch France; ^17^ Brain and Mind Research Institute, Children's Hospital of Eastern Ontario Research Institute Ottawa Ontario Canada; ^18^ Department of Neurology, Heimer Institute for Muscle Research University Hospital Bergmannsheil Bochum Germany

**Keywords:** Charcot–Marie‐Tooth type 4J, developmental delay, juvenile dystonia, juvenile movement disorder, Yunis‐Varón syndrome

## Abstract

**Background:**

Biallelic *FIG4* variants classically cause distinct conditions characterized by peripheral neuropathy (Charcot‐Marie‐Tooth disease type 4J) or skeletal dysplasia (Yunis‐Varón syndrome). However, central nervous system involvement is increasingly recognized. The prevalence and specific characteristics of movement disorders within *FIG4*‐related disease remain poorly characterized, prompting a need to redefine its clinical continuum.

**Objective:**

To delineate the phenotypic spectrum of *FIG4*‐related disorders beyond established entities (Yunis‐Varón syndrome, Charcot‐Marie‐Tooth disease type 4J, bilateral temporooccipital polymicrogyria, and amyotrophic lateral sclerosis type 11) using an international multicentre cohort.

**Methods:**

We retrospectively analyzed clinical, genetic, and neurophysiological data from patients with biallelic *FIG4* variants to redefine genotype‐phenotype correlations, focusing on motor development, cognitive status, and movement disorders.

**Results:**

Evaluating 14 pediatric *FIG4* patients revealed a continuous phenotypic spectrum rather than the clinical entities previously described in the literature. Instead of the typical peripheral neuropathy classically associated with pathogenic *FIG4* variants, the predominant clinical picture in this age group was a severe neurodevelopmental disorder. Patients showed profound motor and cognitive delay, with a subset additionally displaying distinct movement abnormalities such as dystonia and bradykinesia.

**Conclusion:**

Loss of functional FIG4 causes a broader and more severe disease spectrum with central nervous system involvement, including intellectual disability and movement abnormalities such as dystonia and bradykinesia. Recognizing these features should prompt clinicians to consider *FIG4*‐associated disorders, allowing for optimized patient care and tailored therapeutic strategies.

## 1 Introduction

The tight regulation of intracellular vesicle trafficking and endolysosomal homeostasis is fundamental to neuronal survival. A critical component of this machinery is the phosphoinositide phosphatase FIG4, encoded by the *FIG4* gene on chromosome 6q21. Together with the scaffold protein VAC14 and the kinase PIKFYVE, FIG4 forms a ternary complex that regulates the synthesis and turnover of phosphatidylinositol 3,5‐bisphosphate [PI(3,5)P2] [1, 2]. Deficiency in FIG4 function leads to depleted PI(3,5)P2 levels, causing the accumulation of enlarged, vacuolated endolysosomes in neurons and Schwann cells, a cellular hallmark that precipitates progressive neurodegeneration in both the peripheral and central nervous systems [3, 4, 5]. The extent to which cellular FIG4 function is disrupted depends on mutation type and position, resulting in heterogeneous clinical phenotypes and disease trajectories: Complete loss of protein function caused by biallelic null variants results in Yunis‐Varón syndrome (YVS; MIM: 216340), a severe, often lethal disorder characterized by cleidocranial dysplasia and global central nervous system (CNS) involvement. Partial loss of function of FIG4 underlies Charcot‐Marie‐Tooth disease type 4J (CMT4J; MIM: 611228), which most commonly results from compound heterozygosity involving the frequent p.Ile41Thr missense variant and a null allele [6, 7, 8]. Clinically, CMT4J is a severe, early‐onset motor and sensory neuropathy with distinct features of both axonal loss and demyelination, often mimicking acquired inflammatory neuropathies due to non‐uniform conduction slowing and conduction blocks [5, 8]. Recent studies also describe a phenotypic combination of CMT4J and parkinsonism particularly in adulthood (not yet listed in OMIM) suggesting a vulnerability in the neuronal maintenance of dopaminergic signaling pathways in FIG4 deficiency [7, 9]. The description of bilateral temporooccipital polymicrogyria (BTOP, MIM: 612691) as a *FIG4*‐associated phenotype is based on one large consanguineous Moroccan family with affected individuals presenting with complex (temporal lobe) epilepsy, psychiatric symptoms and behavioral abnormalities caused by a homozygous missense variant in *FIG4* (p.Asp783Val) leading to a partial loss of protein function [10, 11]. In addition, heterozygous *FIG4* variants were reported as risk factors for the development of adult‐onset amyotrophic lateral sclerosis type 11 (ALS11; MIM: 612577), highlighting the critical role of FIG4 also in motor neuron maintenance and survival [12].

However, recent studies have significantly expanded the phenotypic spectrum beyond the “pure” phenotypes associated with pathogenic recessive *FIG4* variants. It is now increasingly recognized that such variants may lead to a continuum of phenotypes that bridge CMT4J and YVS, often involving significant CNS manifestations such as cortical atrophy, cerebellar signs, and cognitive impairment [7]. So far, ALS11 and a combination of CMT4J and parkinsonism have only been described in adults. All *FIG4*‐associated diseases are classified as rare (often with private variants), and their clinical severity ranges widely, from mild clinical signs to severe disability [6].

Despite an improved understanding of the clinical spectrum and genotype–phenotype correlations, the full spectrum of pediatric phenotypes remains incompletely characterized to this date. Recent studies have highlighted the variability in disease progression and the need for robust clinical outcome measures, especially in light of emerging gene therapy approaches [7, 8].

In this study, we present the clinical and genetic characteristics of a cohort of 14 pediatric patients with *FIG4*‐associated neurological phenotypes. By detailing the spectrum of peripheral and central nervous system involvement in this neurodevelopmental group, we aim to further refine genotype–phenotype correlations and thus facilitate diagnostic recognition of this multifaceted disorder.

## 2 Materials and Methods

### 2.1 Patient Cohort

Through a retrospective international multicenter study, we collected epidemiological, clinical, and molecular genetic data of 14 pediatric patients harboring biallelic *FIG4* variants. These patients were collected via ESNEK (Surveillance for Rare Neurological Disorders during Childhood) as an e‐mail‐based recruitment tool [13] and preexisting international collaborations. Inclusion criteria comprised biallelic variants in the *FIG4* gene along with neurological features; exclusion criteria included monoallelic *FIG4* variants, lack of specification regarding the exact variants, or insufficient clinical data.

Patients were examined during routine care in their respective clinics by child neurologists with long‐term experience in neuromuscular diseases and movement disorders in childhood. All clinical data were then collected by using standardized case report forms. To avoid semantic redundancy, the data were collected based on Human Phenotype Ontology (HPO) terms [14].

### 2.2 Ethical Considerations

The study received institutional ethics board approval from the Ethics Committee of the Medical Faculty of the University of Duisburg‐Essen (25‐12477‐BO).

## 3 Results

### 3.1 Epidemiology

The cohort consisted of 14 patients from 11 unrelated families. One family (Family 5) had affected twins, while a second family (Family 10) had three brothers with genetically confirmed *FIG4* variants. The other families reported one affected child each. Nine (64.3%) patients were male and five (35.7%) female. Age at onset ranged from birth to 9 years (median 6 months, mean 1.44 years). There were no indications of an antenatal onset (reduced fetal movements, arthrogryposis multiplex congenita or polyhydramnios) in any of the patients. Age at diagnosis ranged from 3 months to 16 years (median 4.13 years, mean 5.93 years). Age at last examination ranged from 4 months to 18.2 years (median 4.96 years, mean 6.93). Twelve (85.7%) patients were of European descent and 2 (14.3%) were of Asian origin, respectively. Seven patients harbored compound heterozygous and seven homozygous *FIG4* variants. Despite the high proportion of homozygous variants, only one family reported consanguinity (Table 1).

**TABLE 1:** Overview of our international cohort of pediatric FIG4 patients: Epidemiological, clinical, and molecular genetic data of 14 patients from 11 different families harboring biallelic *FIG4* variants.

Pt.	Fam.	Age (y)	Gender	Country of origin	Con.	Main clinical manifestation	Variant I						Variant II						Seg.
							DNA level	Protein level	gnomAD allele freq.	CADD score	ACMG criteria	Ref.	DNA level	Protein level	gnomAD allele freq.	CADD score	ACMG criteria	Ref.	
1	1	4.1	Male	Spain	No	Global developmental delay, severe language delay, nystagmus, hypotonia, learned to sit, gait preserved (ataxic), global bradykinesia	c.122T>C	p.Ile41Thr	0.001536	25.5	Pathogenic	[3]	c.995G>C	p.Trp332Ser	0.00000062	32	Likely pathogenic	This re‐port	CH
2	2	14.5	Male	Spain	Yes	Autism, epilepsy, intellectual disability, at 14 years: epileptic decompensation and progressive muscle weakness	c.122T>C	p.Ile41Thr	0.001536	25.5	Pathogenic	[3]							HO
3	3	5.8	Male	United Kingdom	No	Exercise‐induced myalgia, steppage gait, distal upper limb weakness, areflexia, action tremor, (acquired) ophthalmoparesis, dysphagia	c.122T>C	p.Ile41Thr	0.001536	25.5	Pathogenic	[3]	c.904G>A	p.Glu302Lys	0.00000496	26.2	Pathogenic	[6]	CH
4	4	9.9	Female	United Kingdom	No	Global developmental delay, intellectual disability, autism, wheelchair‐dependent, areflexia, scoliosis, multiple joint contractures	c.122T>C	p.Ile41Thr	0.001536	25.5	Pathogenic	[3]	Deletion Exon 2–23		0		Pathogenic	This re‐port	CH
5	5	2.3	Male	Turkey	No	Severe global development delay (no sitting, no standing, no walking), strong suspicion of intellectual disability, absent speech, severe generalized muscle hypotonia, mild dystonia (legs>arms)	c.356T>G	p.Ile119Ser	0	27.1	Likely pathogenic	This re‐port	c.1138G>T	p.Glu380*	0		Pathogenic	This re‐port	CH
6		2.3	Male	Turkey		Severe global development delay (no sitting, no standing, no walking), strong suspicion of intellectual disability, absent speech, severe generalized muscle hypotonia, mild dystonia (legs>arms)	c.356T>G	p.Ile119Ser	0	27.1	Likely pathogenic	This re‐port	c.1138G>T	p.Glu380*	0		Pathogenic	This re‐port	CH
7	6	2.8	Female	Germany	No	Profound global development delay (no sitting, no standing, no walking), strong suspicion of intellectual disability, absent speech, severe generalized muscle hypotonia, mild dystonia (arms), global bradykinesia	c.122T>C	p.Ile41Thr	0.001536	25.5	Pathogenic	[3]	c.863G>A	p.Gly288Asp	0.000001869	26.3	Likely pathogenic	This re‐port	CH
8	7	18.2	Male	Germany	No	Hyporeflexia of lower limbs, difficulties jumping, hypomimia, no heel walking, generalized muscular amyotrophy, distal muscle weakness, pallhypesthesia	c.122T>C	p.Ile41Thr	0.001536	25.5	Pathogenic	[3]							HO
9	8	3.4	Female	India	No	Global development delay, seizures, dystonia, developmental regression, choreoathetosis	c.317A>G	p.Tyr106Cys	0.000001241	26.6	Likely pathogenic	This re‐port							HO
10	9	5.0	Female	India	No	Global development delay, seizures, dystonia, failure to thrive, tetraplegia, hypotonia, visual impairment	c.504C>G	p.Ser168Arg	0.000002481	25.0	Pathogenic	[15]							HO
11	10	12.8	Male	Romania	No	Full‐time wheelchair, generalized muscle weakness, skeletal muscle atrophy, dystonia, bradykinesia, Babinski sign, spasticity, scoliosis, limb joint contracture	c.21del	p.Ile8Serfs*19	0		Pathogenic	This re‐port							HO
12		2.8	Male	Romania	No	Walking with aids/support, gait ataxia, generalized muscle weakness, dystonia, bradykinesia, Babinski sign, spasticity	c.21del	p.Ile8Serfs*19	0		Pathogenic	This re‐port							HO
13		0.8	Male	Romania		Mild motor delay (no rolling over at 10 Months), mild axial hypotonia	c.21del	p.Ile8Serfs*19	0		Pathogenic	This re‐port							HO
14	11	12.0	Female	United Kingdom		Sensory‐motor neuropathy with axonal and demyelinating features, sensory ataxia, distal weakness, scoliosis	c.122T>C	p.Ile41Thr	0.001536	25.5	Pathogenic	This re‐port	c.2065dupA	p.Thr689Asnfs*12	0.000002478		Pathogenic	[16]	CH

Abbreviations: CADD, combined annotation dependent depletion; CH, Compound heterozygous; Con., consanguinity; freq., frequency; gnomAD, genome aggregation database; HO, homozygous; PMID, PubMed unique identifier; Ref., reference; Seg, segregation; y, years.

### 3.2 Clinical Findings

The cohort exhibited a broad and clinically heterogeneous phenotype encompassing neurodevelopmental delay, neuromuscular dysfunction, and movement disorders (Table S1): Global delay constituted a central feature, affecting 10 patients (71.4%), with motor and speech delay equally affected. Fine and gross motor delay was reported in 9 patients (64.3%). Absent speech was documented in 8 patients (57.1%), of whom all were over two years old. Global developmental delay and intellectual disability (ID) were each identified in 7 patients (50%). Developmental regression occurred in 6 patients (42.9%), highlighting a dynamic disease course in a substantial subset. Behavioral comorbidities were frequent, including autism spectrum disorder (4 patients, 28.6%), attention deficit hyperactivity disorder (2 patients, 14.3%), impaired emotional regulation (3 patients, 21.4%), and aggressive or impulsive behavior (3 patients, 21.4%).

Neuromuscular involvement was equally prominent. Abnormal deep tendon reflexes were observed in 10 patients (71.4%), most commonly areflexia (6 patients, 42.9%). Muscle weakness and abnormal muscle tone were each present in 10 patients (71.4%), with hypotonia predominating (8 patients, 57.1%) but hypertonia also reported (4 patients, 28.6%). Weakness was variably distributed, affecting axial (6 patients, 42.9%), proximal (5 patients, 35.7%), distal lower limb (5 patients, 35.7%), distal upper limb (4 patients, 28.6%), and facial musculature (5 patients, 35.7%). Gait abnormalities were highly prevalent (12 patients, 85.7%). Specific findings included unsteady gait in 4 patients (28.6%), while gait ataxia and steppage gait were observed in 3 patients (21.4%) each. Notably, nine patients (64.3%) were unable to walk independently at the time of data acquisition. However, it should be noted that among the four patients characterized as having “never learned to walk independently,” only one was older than three years (Patient 11). Regarding current mobility, only Patient 1 is able to ambulate independently without assistive devices, three patients can ambulate with assistance, and five patients are entirely wheelchair dependent. Only two patients (Patients 3 and 8) had ever achieved the ability to run.

Involuntary movements, present in 7 patients (50%), represented a key feature. Dystonia was most common (5 patients, 35.7%), followed by bradykinesia and gait ataxia (3 patients each, 21.4%). Action tremor occurred in 2 patients (14.3%), while parkinsonism, athetosis, and extrapyramidal dyskinesia were observed in 1 patient (7.1%) each. Pyramidal signs, including hyperreflexia, positive upgoing plantar response (Babinski sign), and spasticity, were each reported in 3 patients (21.4%), with clonus in 1 patient (7.1%).

A progressive course was observed in 8 patients (57.1%), predominantly slow in evolution (7/8 patients, 87.5%), while 2 patients (14.3%) had a non‐progressive course.

Electrophysiological studies (*n* = 8) demonstrated marked heterogeneity with overlapping demyelinating and axonal features. Abnormal sensory nerve conduction velocities were present in 6 patients (75%). Demyelinating motor and sensory neuropathy were each reported in 4 patients (50%), mixed demyelinating‐axonal polyneuropathy in 4 patients (50%), and motor or sensory axonal neuropathy in 3 patients each (37.5%).

Brain MRI (*n* = 9) revealed structural abnormalities in 2 patients (22.2%), characterized by T2 hyperintensities of the cerebral white matter involving the basal ganglia and/or thalamus (Figure 1). None of the patients showed polymicrogyria or hypoplasia of the corpus callosum and/or cerebellar vermis.

**FIGURE 1:**
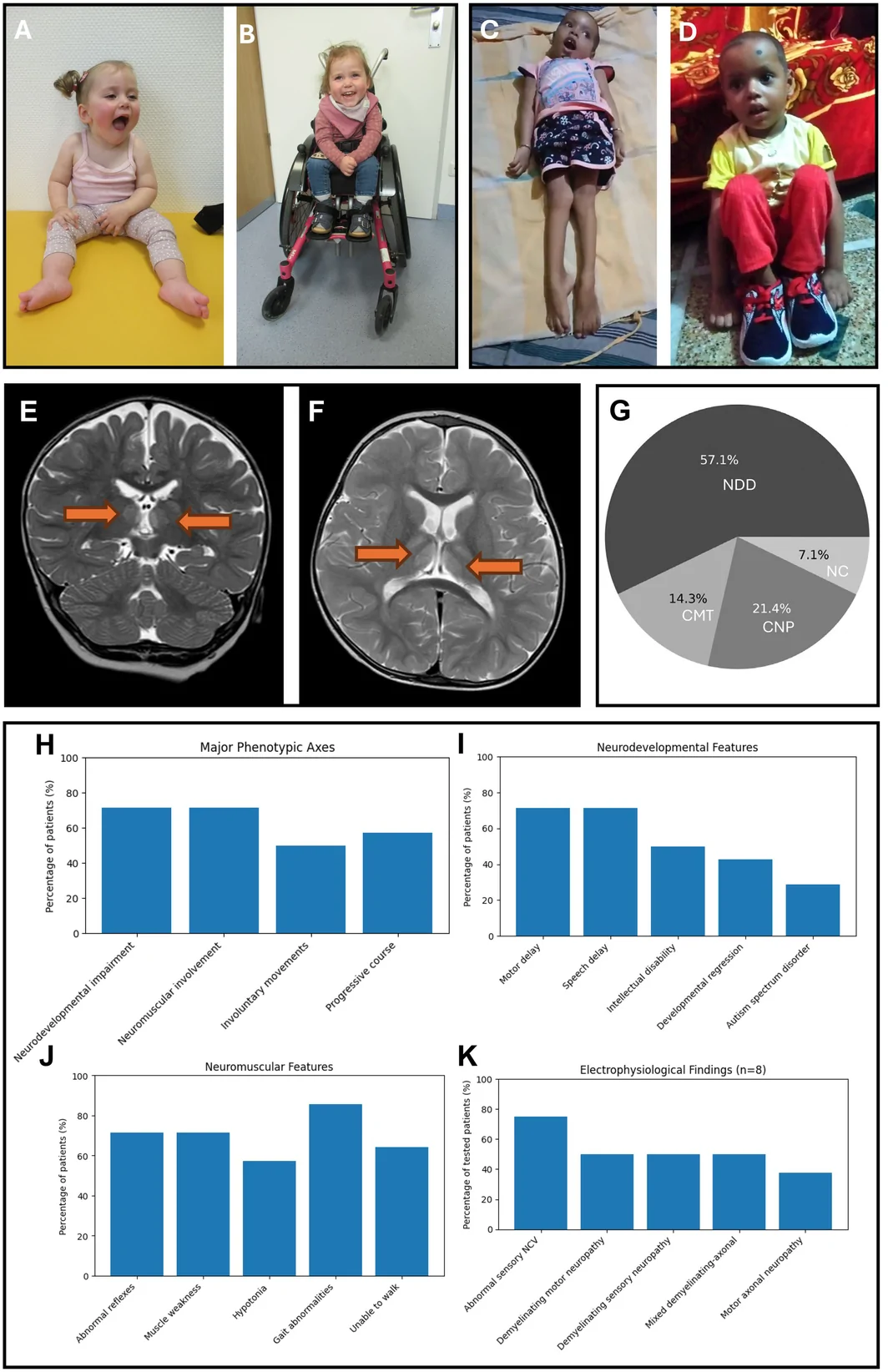
Clinical picture A–D: (A) Female *FIG4*‐Patient (pt. 7) at 2.5 years, not able to sit unsupported. (B) Same patient at the age of 3 years, with her first wheelchair, still not able to sit unsupported, nor stand or walk without help. (C) Female *FIG4* patient (pt. 10) from India at the age of 2 years, showing severe generalized muscle weakness and atrophy in combination with pointed feet. (D) Same patient at the age of 3 years. MRI images E + F: MRI of one of the male *FIG4* twin patients (pt. 5), at the age of 16 months showing an increased coronary (E) and axial (F) T2 signal of both thalami, consistent with the patient's dystonic movement disorder. (G) Pie chart: Dark Gray (57.1%): Neurodevelopmental‐dominant phenotype (NDD, *n* = 8), Medium Gray (21.4%): Complex neurological phenotype (CNP, *n* = 3), Light Gray (14.3%): Classical Charcot–Marie‐Tooth phenotype (CMT, *n* = 2), Very Light Gray (7.1%): Not classified (NC, *n* = 1). Bar graphs H–K: (H) Distribution of major phenotypic axes, (I) Distribution of Neurodevelopmental features, (J) Distribution of neuromuscular features, (K) Distribution of electrophysiological findings (*n* = 8).

Additional systemic features included seizures (generalized and focal) in 2 patients (14.3%), feeding difficulties in 5 patients (35.7%), with chewing difficulties in 4 patients (28.6%), poor swallowing (Dysphagia) in 3 patients (21.4%) and poor suck in 2 patients (14.3%). Skeletal abnormalities including scoliosis were noted in 4 patients (28.6%), foot deformities such as pes cavus in 2 patients (14.3%) and pes planus in 1 patient (7.1%), as well as dysmorphic facial features in 3 patients (21.4%). No cardiac or respiratory involvement was observed (Table S1). Phenotypic stratification revealed three principal clinical patterns: (i) a neurodevelopmental‐dominant phenotype in 8 patients (57.1%), frequently accompanied by movement disorder; (ii) a classical CMT‐like neuropathic phenotype in 2 patients (14.3%), and (iii) a complex neurological phenotype in 3 patients (21.4%) combining severe neuropathy with movement disorder and additional features such as ophthalmoparesis and dysphagia. One patient could not be assessed due to his very young age (patient 13). Figure 1 illustrates representative clinical and neuroimaging findings.

## 4 Phenotype–Genotype Correlations

In our cohort of 14 pediatric patients harboring biallelic pathogenic *FIG4* variants (Figure 2), we identified distinct genotype‐phenotype associations. The recurrent missense variant c.122T>C (p.Ile41Thr) was the most frequent allele, identified in 7 out of 14 patients (50%), irrespective of ethnic origin (Table 1). Zygosity of the p.Ile41Thr variant correlated with differences in clinical severity. Patients homozygous for p.Ile41Thr (*n* = 2; Patients 2 and 8) presented with a milder phenotype and slower disease progression; Patient 8 retained the ability to run at 18 years of age, and Patient 2 remained ambulatory with support at 14 years. Patients harboring the p.Ile41Thr variant in a compound heterozygous state exhibited phenotypic variability. When p.Ile41Thr was combined with a large deletion of exons 2–23 (Patient 4) or the p.Gly288Asp substitution (Patient 7), both individuals became full‐time wheelchair users in early childhood (at ages 9 and 2 years, respectively). In contrast, Patient 1 (p.Ile41Thr/p.Trp332Ser) remained ambulatory at four years of age. Patients presenting with variants other than the p.Ile41Thr allele generally presented with earlier onset and more rapid progression. The frameshift variant c.21del (p.Ile8Serfs*19) was found in a homozygous state in three siblings (Patients 11, 12, 13). The oldest sibling in this family (Patient 11) lost ambulation by 8.5 years, required a full‐time wheelchair by age 12, and presented with severe intellectual disability. Severe intellectual disability was exclusively observed in patients with homozygous truncating mutations or compound heterozygous variants associated with an overall severe disease course.

**FIGURE 2:**
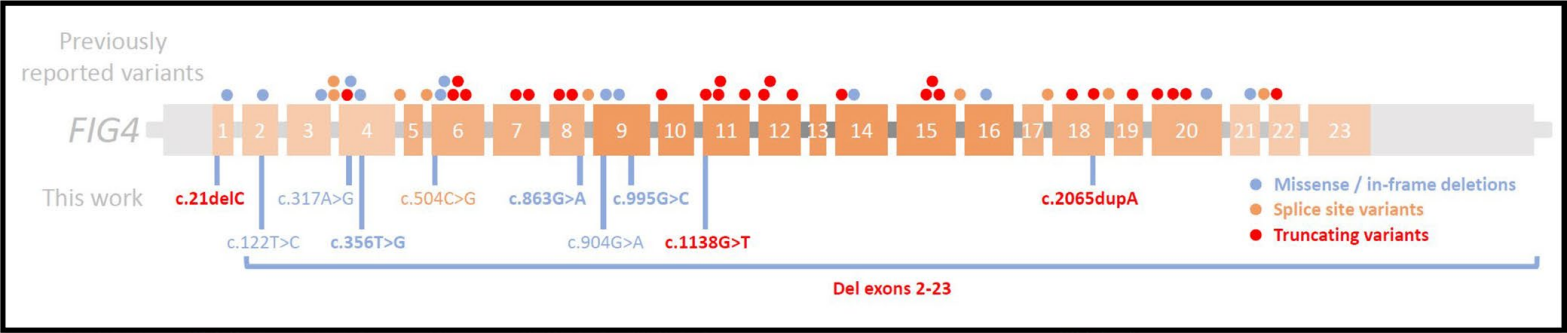
Schematic representation of the *FIG4* gene illustrating the location of previously reported and novel variants (bold) detected in our cohort.

## 5 Discussion

In this international multicentre cohort of 14 pediatric patients with biallelic pathogenic *FIG4* variants, we demonstrate that childhood FIG4 deficiency represents a continuous neurodevelopmental‐neuromuscular spectrum rather than discrete clinical entities. Our data expand the established framework of Charcot–Marie–Tooth disease type 4J (CMT4J) and Yunis‐Varón syndrome (YVS), defining a severe neurodevelopmental phenotype characterized by early‐onset global developmental impairment, intellectual disability, and a prominent movement disorder component.

While FIG4‐associated disease has historically been conceptualized primarily as a peripheral neuropathy (CMT4J), the predominant clinical picture in our pediatric cohort was a neurodevelopmental phenotype. Failure to achieve motor milestones, absent or severely delayed speech, and intellectual disability were evident early in life, often preceding overt neuropathic features. This aligns with recent reports of adult patients carrying biallelic *FIG4* mutations who presented with a combination of sensorimotor peripheral neuropathy and various central nervous system (CNS) pathologies [7], as well as data from the Inherited Neuropathy Consortium (INC) highlighting cognitive difficulties in CMT4J [8].

Comparing our cohort with the INC cohort (*n* = 19; 5 adults and 14 pediatric patients), several key clinical distinctions emerge. Notably, our patients were significantly younger at the time of examination than the INC pediatric subset (mean age 6.93 vs. 10.9 years). While motor developmental delay was prevalent across both groups, the phenotypic emphasis differed: the INC cohort predominantly exhibited distal and asymmetric muscle weakness—features typical of a classical neuropathy presentation. In contrast, these specific deficits were less prominent in our cohort, which was instead characterized more broadly by severe developmental delays. Furthermore, while cognitive deficits were highly frequent in both cohorts, respiratory impairment was exclusively reported in the INC cohort, likely reflecting the older age and progressive nature of the disease in that population. Regarding genotype–phenotype correlations, both cohorts consistently demonstrated a milder clinical trajectory in patients homozygous for the *FIG4* p.Ile41Thr variant.

Our clinical findings suggest that FIG4 dysfunction interferes not only with axonal maintenance but also with early neuronal circuit maturation. Given the central role of FIG4 in regulating phosphatidylinositol 3,5‐bisphosphate [PI(3,5)P_2_] within the endolysosomal pathway, impaired vesicular trafficking and autophagic flux during critical developmental windows may contribute to disrupted synaptic development and cortical network formation. Thus, in pediatric patients, FIG4 deficiency should be considered as a disorder at the intersection of neurodevelopment and neurodegeneration, in keeping with similar observations in closely related neurodevelopmental and neurodegenerative disorders affecting endolysosomal and autolysosomal pathways, such as VAC14 (a binding partner of FIG4) [17] as well as recessive mutations in the tethering factor EPG5 [18, 19].

A key clinical observation of our study is the unexpectedly high prevalence of movement disorders, present in half of the cohort. Dystonia and bradykinesia were the most frequent manifestations and, in several cases, contributed substantially to overall disability. Although adult‐onset parkinsonism in FIG4‐associated disease has been previously described in multiple cases [7, 9, 20], extrapyramidal symptoms in children are likely under‐recognized in the literature. To date, only a few pediatric patients have been reported with infant‐onset dystonia and cognitive impairment. The frequent occurrence of dystonia and bradykinesia, together with basal ganglia and thalamic signal abnormalities in a subset of our patients, points to a selective vulnerability of subcortical motor networks. While adult FIG4 cohorts showed cerebral MRI findings of global brain atrophy, mild leukoencephalopathy, and signal changes in the pons, our cohort demonstrated signal increases in the thalamus and basal ganglia, similar to previously reported T2 hyperintensities in the olivary nuclei in accordance with FIG4‐related leukoencephalopathy [15, 21]. These summarized clinical findings align FIG4 malfunction with other disorders of endolysosomal dysfunction that combine peripheral neuropathy and central movement abnormalities, such as Niemann‐Pick type C [22], complex spastic paraplegias (SPG11 and SPG15) [23], or Krabbe disease [24]. Importantly, the recognition of dystonia and bradykinesia as cardinal features has immediate clinical implications, supporting the early involvement of movement disorder specialists and the consideration of symptomatic pharmacological interventions.

At first glance, our integrated genetic and clinical data support a dosage‐dependent model of FIG4‐related disease onset and progression: truncating *FIG4* variants are generally associated with earlier onset and more severe disease manifestations compared with missense variants, supporting a loss‐of‐function mechanism as a major determinant of clinical severity. However, the clinical spectrum cannot be fully explained by a simple missense‐versus‐truncating dichotomy. Patients harboring compound heterozygous genotypes consisting of one missense and one presumed null allele exhibited an intermediate phenotype, indicating that residual function contributed by the missense allele may partially modulate disease severity. Substantial variability was observed within this group of compound heterozygotes; given the shared presence of the recurrent p.Ile41Thr variant in several patients, these differences strongly suggest that the biochemical properties of the second missense variant critically influence the clinical course. The extent of variant‐induced protein misfolding and its consequences for residual enzymatic activity and protein stability are likely key modulators of phenotypic severity. In this context, particularly deleterious missense variants may cause pronounced misfolding and potential aggregate formation, thereby not only reducing functional protein levels but possibly exerting an additional toxic gain‐of‐function effect. Because FIG4 operates within the PIKFYVE‐VAC14‐FIG4 complex, pathogenic variants may differentially affect enzymatic activity, protein stability, or complex assembly, in turn impacting cellular PI(3,5)P2 homeostasis. In light of all these aspects, a threshold model of residual enzyme activity may apply: minimal remaining function may suffice for PI(3,5)P2 synthesis and turnover for peripheral nerve maintenance but be inadequate for normal brain development. The confinement of severe intellectual disability to patients with predicted null or functionally severe genotypes strongly supports this concept. Comparative functional analyses of distinct missense variants in well‐controlled cellular models are required to substantiate this hypothesis and establish a robust, biochemically informed genotype–phenotype correlation.

Overall, our clinical findings support a concept of three emerging principal phenotypic patterns: a neurodevelopmental‐dominant phenotype frequently accompanied by a movement disorder, a classical CMT‐like neuropathic phenotype, and a complex phenotype combining severe neuropathy with central nervous system involvement. These patterns likely represent points along a biological continuum rather than discrete syndromes. Thus, our data broaden the diagnostic spectrum in which *FIG4* variants should be considered. Testing should be contemplated in infants with hypotonia and global developmental delay, in children with early dystonia or bradykinesia with or without neuropathic signs, and in complex CMT phenotypes accompanied by intellectual disability. This expanding phenotype also has implications for emerging therapeutic approaches; for instance, preclinical studies have demonstrated the benefit of AAV‐mediated FIG4 replacement in CMT4J mouse models [4]. Our data suggest that early therapeutic intervention may be particularly relevant in severe neurodevelopmental phenotypes, where trajectories are affected from infancy. As phenotypic stratification suggests that CNS and peripheral nervous system (PNS) involvement do not necessarily progress in parallel, future clinical trials will need to incorporate holistic outcome measures capturing both peripheral neuropathy and central manifestations, including movement disorders and cognitive function. Ultimately, definitive conclusions can only be drawn from larger cohorts, which remains a significant challenge in the study of rare diseases, particularly regarding pediatric subgroups.

## 6 Limitations

The interpretation of our findings is primarily constrained by the retrospective study design and the modest sample size, which precludes formal statistical modeling of genotype–phenotype correlations. Furthermore, the absence of standardized scales/instruments, neuropsychological assessments, and the lack of uniform neuroimaging and electrophysiological protocols across centers limit the accuracy of our clinical data. Nevertheless, the high consistency of the observed clinical patterns across multiple independent international centers strongly supports the robustness of the proposed phenotypic continuum. While our cohort provides detailed phenotyping of movement disorder manifestations, a limitation of this retrospective study is the lack of standardized data regarding specific management strategies and long‐term treatment outcomes.

## 7 Conclusion

Our study redefines childhood‐onset *FIG4*‐associated disease as a broad neurodevelopmental‐neuromuscular spectrum characterized by prominent central nervous system involvement. Notably, dystonia and bradykinesia emerge as cardinal features of a severe neurodevelopmental phenotype, challenging the traditional view of the disease as primarily an isolated peripheral neuropathy. The recognition of this expanded clinical continuum has immediate implications for diagnostic workups, genetic counseling, and the design of future therapeutic interventions.

Based on our findings, we propose the following five clinical recommendations to guide the management of pediatric *FIG4* deficiency:

1. Broaden Diagnostic Screening: Consider *FIG4* genetic testing in infants and children presenting with unexplained global developmental delay, hypotonia, and early‐onset movement disorders, even in the absence of overt peripheral neuropathic signs.
2. Early Movement Disorder Assessment: Actively screen for and monitor extrapyramidal symptoms (particularly dystonia and bradykinesia) in all patients with confirmed *FIG4* variants to facilitate the early introduction of targeted symptomatic pharmacological interventions.
3. Targeted Neuroimaging: Include a specific evaluation of the basal ganglia and thalamus (e.g., assessing for T2 hyperintensities) in the structural MRI protocols of suspected patients, to identify the selective vulnerability of subcortical motor networks.
4. Multidisciplinary Care: Establish a coordinated, multidisciplinary care approach involving child neurologists, movement disorder specialists, and neuropsychologists to address both the central (cognitive and extrapyramidal) and peripheral (neuromuscular) disease manifestations simultaneously. Establishing targeted therapeutic protocols for the complex movement disorders in this specific population remains an important goal for future prospective studies.
5. Holistic Clinical Trial Design: For emerging therapies, such as AAV‐mediated gene replacement, incorporate robust, multi‐domain outcome measures that systematically evaluate cognitive development and movement disorder severity alongside the classical peripheral neuropathy scale.

## Author Contributions

A.R., A.G. and U.S.‐S. designed this study. A.G., H.S.D., H.J., D.N.‐d.B. and A.R. wrote the manuscript. T.R., P.M., G.W., H.S.D., D.‐G.B., C.R., V.K.G., V.M.S., C.M.A., A.D., L.C.‐G., D.M., M.V., V.S.R., B.S., L.M. and D.N.‐d.B. provided clinical and/or genetic data. A.R., H.K., J.B. and U.S.‐S. helped with interpreting the data. All authors have read and agreed to the published version of the manuscript.

## Funding

A.G. acknowledges the support from the Mediment Program and Josepha und Charlotte von Siebold Habilitandinnen‐Förderprogramm (both of the Medical Faculty of the University Duisburg‐Essen). D.N.d.B. was supported by the Miguel Servet program from Instituto de Salud Carlos III, Spain (CP22/00141). Parts of this study were funded by the European Regional Development Fund (ERDF) (B2B‐RARE grant to A.R. and U.S.‐S.). A.R. and U.S.‐S. moreover acknowledge the funding by the German Society of Muscular Diseases (DGM).

## Consent

Written informed consent was obtained from each patient or the parents of the patients < 18 years old. This study was conducted in accordance with the principles of the Declaration of Helsinki.

## Conflicts of Interest

The authors declare no conflicts of interest.

## Supporting information


**Table S1:** Overview of clinical manifestations in pediatric FIG4 patients. The table summarizes the clinical phenotypes observed in our patient cohort, organized by symptom category. Frequencies are provided in absolute numbers (*n*) and percentages (%). Symptoms are detailed using the corresponding Human Phenotype Ontology (HPO) terms and identification numbers.

## Data Availability

The original contributions presented in this study are included in the article. Further inquiries can be directed to the corresponding author.
