# Supplementary material for: Clinical Variability and Genotype Driven Outcomes in an International Cohort of 14 Pediatric FIG4 Patients
Source: Ann Clin Transl Neurol. 2026 Nov 3:10.1002/acn3.70561. Online ahead of print. doi: 10.1002/acn3.70561 (PMC13729235; doi:10.1002/acn3.70561)
Supplement: Supplementary file 1 [file ACN3-9999-0-s001.docx]

| **Category** | **Symptom (HPO)** | **n** | **%** |
| --- | --- | --- | --- |
| **Development** | Neuro-developmental abnormality (0012759) | 10 | 71.4% |
|  | Global developmental delay (0001263) | 7 | 50.0% |
|  | Developmental regression (0002376) | 6 | 42.9% |
|  | Abnormality of prenatal development or birth (0001197) | 1 | 7.1% |
| **Speech** | Delayed speech and language development (0000750) | 10 | 71.4% |
|  | Absent speech (0001344) | 8 | 57.1% |
| **Motor** | Motor delay (0001270) | 10 | 71.4% |
|  | Delayed fine motor development (0010862) | 9 | 64.3% |
|  | Delayed gross motor development (0002194) | 9 | 64.3% |
|  | Decreased fetal movement (0001558) | 1 | 7.1% |
| **Intellectual disability** | Intellectual disability (ID) (0001249) | 7 | 50.0% |
| **Behaviour** | Autism (0000717) | 4 | 28.6% |
|  | Abnormal aggressive, impulsive or violent behaviour (0006919) | 3 | 21.4% |
|  | Abnormal emotion/affect behaviour (0100851) | 3 | 21.4% |
|  | Behavioral abnormality (psychiatric disorders) (0000708) | 3 | 21.4% |
|  | Attention deficit hyperactivity disorder (ADHD) (0007018) | 2 | 14.3% |
|  | Sleep disturbance (0002360) | 2 | 14.3% |
| **Epileptic seizures** | EEG abnormality (0002353) | 2 | 14.3% |
|  | Epileptic seizures (0001250) | 2 | 14.3% |
|  | Generalized seizures (0002197) | 2 | 14.3% |
|  | Generalized tonic-clonic seizures (0002069) | 2 | 14.3% |
|  | Epileptiform EEG discharges (0011182) | 1 | 7.1% |
|  | Focal seizures (0007359) | 1 | 7.1% |
|  | Status epilepticus (0002133) | 1 | 7.1% |
| **Myalgia** | Gastronemius mylagia (0031921) | 3 | 21.4% |
|  | Myalgia (0003326) | 3 | 21.4% |
|  | EMG abnormality (0003457) | 2 | 14.3% |
|  | Exercise-induced myalgia (0003738) | 2 | 14.3% |
|  | Muscle spasm (muscle cramps) (0003394) | 1 | 7.1% |
| **Dysauto-nomia** | Constipation (0002019) | 3 | 21.4% |
|  | Dysautonomia (0002459) | 2 | 14.3% |
|  | Autonomic bladder dysfunction (0005341) | 1 | 7.1% |
| **Gastro-entero-logical** | Gastro-enterological symptoms | 4 | 28.6% |
|  | Feeding difficulties (0011968) | 5 | 35.7% |
|  | Chewing difficulty (0005216) | 4 | 28.6% |
|  | Poor swallowing (Dysphagia) (0002015) | 3 | 21.4% |
|  | Poor suck (0002033) | 2 | 14.3% |
|  | Nasogastric tube feeding in infancy (0011470) | 1 | 7.1% |
|  | Nausea and vomiting (0002017) | 1 | 7.1% |
| **Endocrine** | Abnormality of body height (0000002) | 3 | 21.4% |
|  | Short stature (0004322) | 3 | 21.4% |
|  | Abnormal circulating hormone level (0003117) | 1 | 7.1% |
|  | Abnormality of the endocrine system (0000818) | 1 | 7.1% |
| **Abnormal gait** | Inability to walk (0002540) | 9 | 64.3% |
|  | Gait abnormalities (0001288) | 12 | 85.7% |
|  | Unsteady gait (Gait instability) (0002317) | 4 | 28.6% |
|  | Gait ataxia (0002066) | 3 | 21.4% |
|  | Steppage gait (0003376) | 3 | 21.4% |
| **Muscle weakness** | Muscle weakness (0001324) | 10 | 71.4% |
|  | Axial muscle weakness (0003327) | 6 | 42.9% |
|  | Distal lower limb muscle weakness (0009053) | 5 | 35.7% |
|  | Generalized muscle weakness (0003324) | 5 | 35.7% |
|  | Proximal muscle weakness (0003701) | 5 | 35.7% |
|  | Proximal muscle weakness in lower limbs (0008994) | 5 | 35.7% |
|  | Proximal muscle weakness in upper limbs (0008997) | 5 | 35.7% |
|  | Weakness of facial musculature (0030319) | 5 | 35.7% |
|  | Distal muscle weakness (0002460) | 4 | 28.6% |
|  | Distal upper limb muscle weakness (0008959) | 4 | 28.6% |
|  | Pelvic girdle muscle weakness (0003749) | 4 | 28.6% |
|  | Fatigable weakness (0003473) | 3 | 21.4% |
| **Muscle size** | Abnormality of muscle size (0030236) | 2 | 14.3% |
|  | Distal lower limb amyotrophy (0008944) | 2 | 14.3% |
|  | Skeletal muscle atrophy | 2 | 14.3% |
|  | Distal upper limb amyotrophy (0007149) | 1 | 7.1% |
|  | Generalized amytrophy (0003700) | 1 | 7.1% |
|  | Proximal lower limb amyotrophy (0008956) | 1 | 7.1% |
|  | Proximal upper limb amyotrophy (0008948) | 1 | 7.1% |
| **Muscle tone** | Abnormal muscle tone (0003808) | 9 | 64.3% |
|  | Hypotonia (0001252) | 8 | 57.1% |
|  | Muscular hypotonia of the trunk (0008936) | 6 | 42.9% |
|  | Facial hypotonia (0000297) | 5 | 35.7% |
|  | Generalized hypotonia (0001290) | 5 | 35.7% |
|  | Hypertonia (0001276) | 4 | 28.6% |
|  | Neonatal hypotonia (0001319) | 1 | 7.1% |
| **Tendon reflexes** | Abnormal deep tendon reflexes (0031826) | 10 | 71.4% |
|  | Areflexia (0001284) | 6 | 42.9% |
|  | Hyperreflexia (0001347) | 3 | 21.4% |
|  | Reduced tendon reflexes (0001315) | 2 | 14.3% |
| **Pyramidal signs** | Abnormal pyramidal signs (0007256) | 3 | 21.4% |
|  | Babinski sign (0003487) | 3 | 21.4% |
|  | Spasticity (0001257) | 3 | 21.4% |
|  | Clonus (0002169) | 1 | 7.1% |
|  | Spastic hemiparesis (0011099) | 1 | 7.1% |
|  | Spastic tetraparesis (0001285) | 1 | 7.1% |
| **Involuntary movements** | Involuntary movements (0004305) | 7 | 50.0% |
|  | Dystonia (0001332) | 5 | 35.7% |
|  | Bradykinesia (0002067) | 3 | 21.4% |
|  | Action tremor (0002345) | 2 | 14.3% |
|  | Tremor (0001337) | 2 | 14.3% |
|  | Athetosis (0002305) | 1 | 7.1% |
|  | Extra-pyramidal dyskinesia (0007308) | 1 | 7.1% |
|  | Parkinsonism (0001300) | 1 | 7.1% |
|  | Fasciculations (0002380) | 2 | 14.3% |
| **Sensory** | Impaired proprioception (0010831) | 2 | 14.3% |
|  | Sensory impairment (0003474) | 2 | 14.3% |
|  | Impaired pain sensation (0007328) | 1 | 7.1% |
|  | Impaired vibratory sensation (0002495) | 1 | 7.1% |
| **Cranial nerves** | Abnormality of the eye (0000478) | 4 | 28.6% |
|  | Strabismus (0000486) | 2 | 14.3% |
|  | Ophthalmo-paresis (0000597) | 1 | 7.1% |
|  | Abnormality of the cranial nerves (0001291) | 1 | 7.1% |
|  | Endocrinological symptoms or abnormal test results | 1 | 7.1% |
| **Skeletal** | Scoliosis (0002650) | 4 | 28.6% |
|  | Abnormality of the face (0000271) | 3 | 21.4% |
|  | Abnormality of the foot (0001760) | 3 | 21.4% |
|  | Dysmorphic facial features (0001999) | 3 | 21.4% |
|  | Abnormality of joint mobility (0011729) | 2 | 14.3% |
|  | Abnormality of the skeletal system (0000924) | 2 | 14.3% |
|  | Abnormality of the vertebral column (0000925) | 2 | 14.3% |
|  | Contractures of the ankles (0006466) | 2 | 14.3% |
|  | Finger joint hypermobility (0006094) | 2 | 14.3% |
|  | Joint hypermobility (001382) | 2 | 14.3% |
|  | Pes cavus (0001761) | 2 | 14.3% |
|  | Thoracic kyphosis (0002942) | 2 | 14.3% |
|  | Abnormal skull morphology (0000929) | 1 | 7.1% |
|  | Abnormality of the nose (0000366) | 1 | 7.1% |
|  | Abnormality of the periungual region (0100803) | 1 | 7.1% |
|  | Contracture of knees (0006380) | 1 | 7.1% |
|  | Contratcure of hips (0003273) | 1 | 7.1% |
|  | Elbow flexion contracture (0002987) | 1 | 7.1% |
|  | Interphalangeal joint contracture of inger (001220) | 1 | 7.1% |
|  | Joint subluxation (0032153) | 1 | 7.1% |
|  | Knee joint hypermobility (0045086) | 1 | 7.1% |
|  | Limb joint contracture (0003121) | 1 | 7.1% |
|  | Microcephaly (0000252) | 1 | 7.1% |
|  | Pes planus (0001763) | 1 | 7.1% |
|  | Talipes equinovarus (=clubfoot) (0001762) | 1 | 7.1% |
